# Psychiatric Morbidity Among the Patients of First Ever Ischaemic Stroke

**DOI:** 10.1192/bjo.2022.201

**Published:** 2022-06-20

**Authors:** Muhammad Sayed Inam

**Affiliations:** Sylhet MAG Osmani Medical College, Sylhet, Bangladesh

## Abstract

**Aims:**

To evaluate psychiatric morbidity among the patients of first ever ischemic stroke

**Methods:**

This sectional comparative study was carried out in the Department of Psychiatry, Sylhet MAG Osmani Medical College Hospital, Sylhet during the period from 1st July 2013 to 30th June 2014. Sixty six ischaemic stroke patients of first attack between 2 weeks to 2 years of stroke, aged above 18 years irrespective of sex and 66 accompanying healthy person of the patients and other patients without any kind of stroke matching age and sex fulfilling inclusion and exclusion criteria were taken in Group-A and Group-B respectively. Diagnosis of ischaemic stroke was made in these patients by the consultant neurologists reviewing the history, clinical examination and accompanying investigations reports specially CT scan of brain. Psychiatric assessment was done using General Health Questionnaire (GHQ12) as screening tool. All GHQ12 positive cases were evaluated using mental state examination and recorded in a MSE sheet. Diagnosis of psychiatric disorders of all respondents was confirmed by psychiatrist according to DSM-5 criteria.

**Results:**

The patients with ischaemic stroke and control subjects were similar in age [57.6 (SD ± 5.5) years vs 57.1 (SD ± 4.5) years; p > 0.130] and sex [48 (72.7%) male and 18 (27.3%) female vs 45 (68.2%) male and 21 (31.8%) female; p = 0.567]. Comorbid psychiatric disorder was found in 23 (34.8%) patients of ischaemic stroke and 9 (13.6%) control subjects. The comorbid psychiatric disorder was significantly higher in patients of ischaemic stroke than that of control g subjects (p = 0.004). Comorbid specific psychiatric disorders were generalized anxiety disorder in 9 (13.6%) and major depressive disorder in 14 (21.2%) in stroke group; while comorbid specific psychiatric disorders were generalized anxiety disorder in 2 (3.0%) and major depressive disorder in 7 (10.6%) respondents in control group (p < 0.013).

**Conclusion:**

Comorbid psychiatric disorders are quite common among patients with first ever ischaemic stroke in the form of major depressive disorder and generalized anxiety disorder. Therefore, attention should be paid to the anxiety and depressive symptoms in stroke units and try to relieve the patients’ emotional stress and personal suffering, which could improve their neurological outcome.

